# Determination of the Synthetic Cannabinoids JWH-122, JWH-210, UR-144 in Oral Fluid of Consumers by GC-MS and Quantification of Parent Compounds and Metabolites by UHPLC-MS/MS

**DOI:** 10.3390/ijms21249414

**Published:** 2020-12-10

**Authors:** Nunzia La Maida, Manuela Pellegrini, Esther Papaseit, Clara Pérez-Mañá, Lourdes Poyatos, Mireia Ventura, Liliana Galindo, Francesco Paolo Busardò, Simona Pichini, Magí Farré, Emilia Marchei

**Affiliations:** 1 Department of Excellence of Biomedical Science and Public Health, University “Politecnica delle Marche” of Ancona, Via Tronto 71, 60124 Ancona, Italy; n.lamaida@pm.univpm.it (N.L.M.); f.p.busardo@staff.univpm.it (F.P.B.); 2 National Centre on Addiction and Doping, Istituto Superiore di Sanità, V.Le Regina Elena 299, 00161 Rome, Italy; simona.pichini@iss.it (S.P.); emilia.marchei@iss.it (E.M.); 3 Clinical Pharmacology Unit, Hospital Universitari Germans Trias i Pujol and Institut de Recerca Germans Trias i Pujol (HUGTiP-IGTP), 08916 Badalona, Spain; epapaseit.germanstrias@gencat.cat (E.P.); cperezm.mn.ics@gencat.cat (C.P.-M.); lpoyatos@igtp.cat (L.P.); mfarre.germanstrias@gencat.cat (M.F.); 4 Department of Pharmacology, Therapeutics and Toxicology, Universitat Autònoma de Barcelona, 08193 Cerdanyola del Vallés, Spain; 5 Energy Control, Associació Benestar i Desenvolupament, 08012 Barcelona, Spain; mireia@energycontrol.org (M.V.); lg532@cam.ac.uk (L.G.); 6 Department of Psychiatry, University of Cambridge/Cambridgeshire and Peterborough NHS Foundation Trust, Cambridge CB20QQ, UK

**Keywords:** synthetic cannabinoids, JWH-122, JWH-210 and UR-144, oral fluid, GC-MS, UHPLC-HRMS

## Abstract

The consumption of synthetic cannabinoids (SCs) has significantly increased in the last decade and the analysis of SCs and their metabolites in human specimens is gaining interest in clinical and forensic toxicology. A pilot study has been carried out using a combination of an initial last generation gas chromatography-mass spectrometry (GC-MS) screening method for the determination of JWH-122, JWH-210, UR-144) in oral fluid (OF) of consumers and an ultra-high performance liquid chromatography high resolution mass spectrometry (UHPLC-HRMS) confirmatory method for the quantification of the parent compounds and their metabolites in the same biological matrix. OF samples were simply liquid-liquid extracted before injecting in both chromatographic systems. The developed methods have been successfully validated and were linear from limit of quantification (LOQ) to 50 ng/mL OF. Recovery of analytes was always higher than 70% and matrix effect always lower than 15% whereas intra-assay and inter-assay precision and accuracy were always better than 16%. After smoking 1 mg JWH-122 or UR-144 and 3 mg JWH-210, maximum concentration of 4.00–3.14 ng/mL JWH-122, 8.10–7.30 ng/mL JWH-210 ng/mL and 7.40 and 6.81 ng/mL UR-144 were measured by GC-MS and UHPLC-HRMS respectively at 20 min after inhalation. Metabolites of JWH 122 and 210 were quantified in OF by UHPLC-HRMS, while that of UR144 was only detectable in traces. Our results provide for the first time information about disposition of these SCs and their metabolites in consumers OF. Last generation GC-MS has proven useful tool to identify and quantify parent SCs whereas UHPLC-HRMS also confirmed the presence of SCs metabolites in the OF of SCs consumers.

## 1. Introduction

Synthetic cannabinoids (SCs), as indicated by the name, are an ever-expanding family of compounds synthesized in the laboratory for therapeutic and research purposes, including the qualities of endocannabinoids and phytocannabinoids [1].

To date, hundreds of SCs are available on the illicit market being structurally different and categorized into different groups (adamantoylindoles, aminoalkylindoles, benzoylindoles, cyclohexylphenols, dibenzopyrans, naphthoylindoles, naphthylmethylindoles, naphthyl-methylindenes, naphthoylpyrroles, phenylacetylindoles, tetramethylcyclopropyl ketone indoles, quinolinyl ester indoles, and indazole carboxamide compounds) [2]. Although many of them are banned by international laws, web and street illegal markets can provide a huge selection of SCs [3].

In Europe, seizures of new psychoactive substances (NPS) are typically dominated by synthetic cannabinoids which together with synthetic cathinones accounted for 77% NPS seizures [3]. The consumption of SCs has increased in the last decade with a great social incidence, due to the high number of undesirable episodes of intoxications and deaths reported in many countries [1]. Toxic effects such as tachycardia, nausea, somnolence, hypertension, restlessness, and/or agitation have frequently been reported, with fatalities involving myocardial infarction, arrhythmia-related sudden cardiac death and cerebral ischemia [4,5].

An important factor in consumers’ health risks associated with SCs is the variation in ingredients and in quantity of active compounds in herbal smoking mixtures [6,7]. Typically, SCs dosages vary greatly according to the type of active ingredient and their concentrations in smoking mixtures are variables so that to estimate the appropriate amount of herbal mixture to consume can become impossible [7].

Not knowing which compound is consumed and at which dose means that when identification or quantification are required in biological fluid of intoxicated consumers, the analytical challenge involves not only the large range of different compounds and/or metabolites to identify, but also the use of highly sensitive techniques, that allow the determination of the eventually low compounds concentrations. This occurrence is especially striking when alternative non-invasive biological matrices such as OF and hair are investigated in cases of intoxication and fatalities [8,9,10,11].

In this concern, OF has become increasingly popular as alternative biological specimen to blood for the non-invasive detection of parent drugs and metabolites. Drug excretion by salivary glands depends on matrix pH, substance pKa and lipophilicity, and amount of unbound drug in the bloodstream. Highly lipid-soluble drugs, such as SCs, may cross into the oral cavity by passive transcellular diffusion suggesting detection in OF [12].

As above outlined, typical synthetic cannabinoid intake is in the low mg range. Thus, very low concentrations are expected to be found in biological fluids including OF [13]. Determination of SCs in OF has been successfully accomplished by gas chromatography mass spectrometry (GC-MS) [13,14,15], and by liquid chromatography tandem mass spectrometry (LC-MS/MS) [16,17,18,19,20,21,22,23,24]. Scarce data are available about synthetic cannabinoids oral fluid pharmacokinetic properties in humans [20,24,25] and no published method has targeted metabolites yet. As matter of fact, it can be of importance to investigate SCs pharmacokinetics and their disposition into biological fluids and tissues to understanding the onset, magnitude, and duration of pharmacodynamics effects, so as eventual concentrations associated to intoxications.

In this regard, we focused on the development and validation of two analytical methods for SCc determination in OF: a last generation gas chromatography–mass spectrometry (GC-MS) method for an initial target analysis of JWH-122, JWH-210 and UR-144 in consumers OF and then an ultra-high performance liquid chromatography high resolution mass spectrometry (UHPLC-HRMS) method for a complementary measurement of JWH-122, JWH-210, UR-144 and their metabolites time course in consumers OF after a single smoking self-administration. We focused on these particular substances due to their recent spread on the Spanish illegal market, as reported by the same consumers in web fora and to a Spanish non-governmental organization dealing with drug risk reduction, presenting several described adverse effects [26,27,28,29].

## 2. Results

### 2.1. GC-MS, UHPLC-HRMS and Validation Parameters

**Figure 1:**
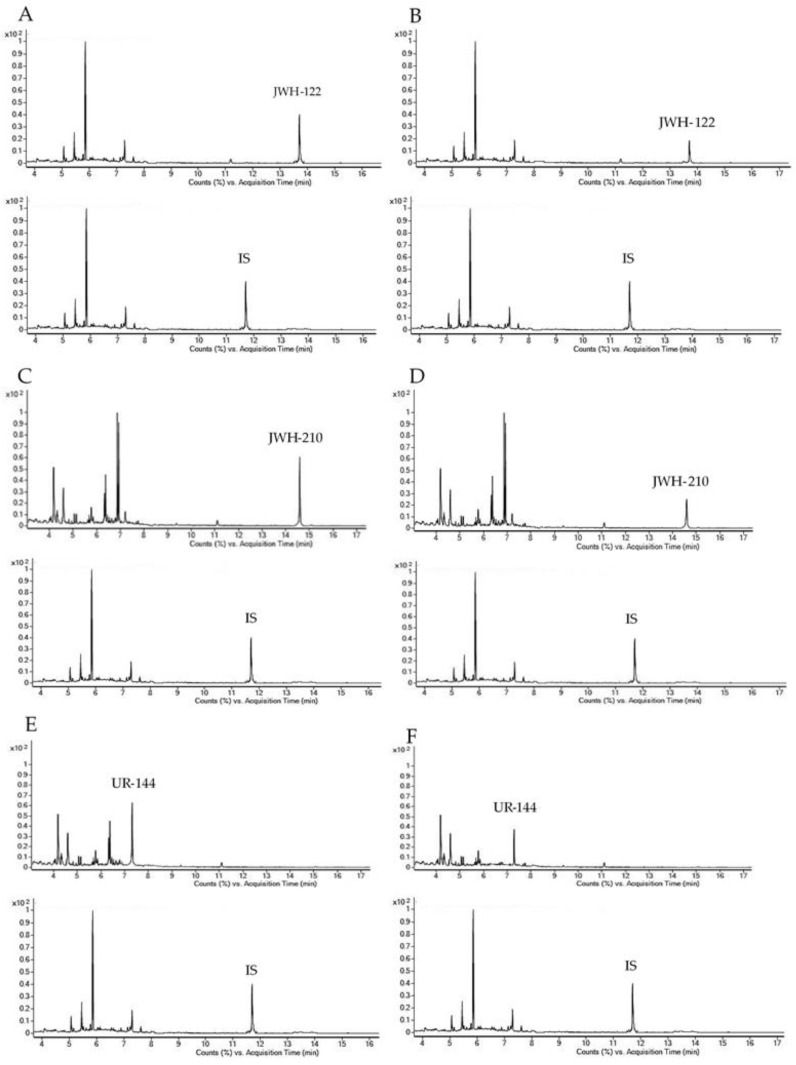
Gas chromatography-mass spectrometry (GC-MS) single ion monitoring chromatograms of 400 µL OF extract spiked with 1.00 ng/mL JWH-122 (**A**), JWH-210 (**C**) and UR-144 (**E**); OF samples consumers containing 4.00 ng/mL JWH-122 (**B**); 8.10 ng/mL JWH-210 (**D**); 7.40 ng/mL UR-144 (**F**). IS, internal standard.

**Figure 2:**
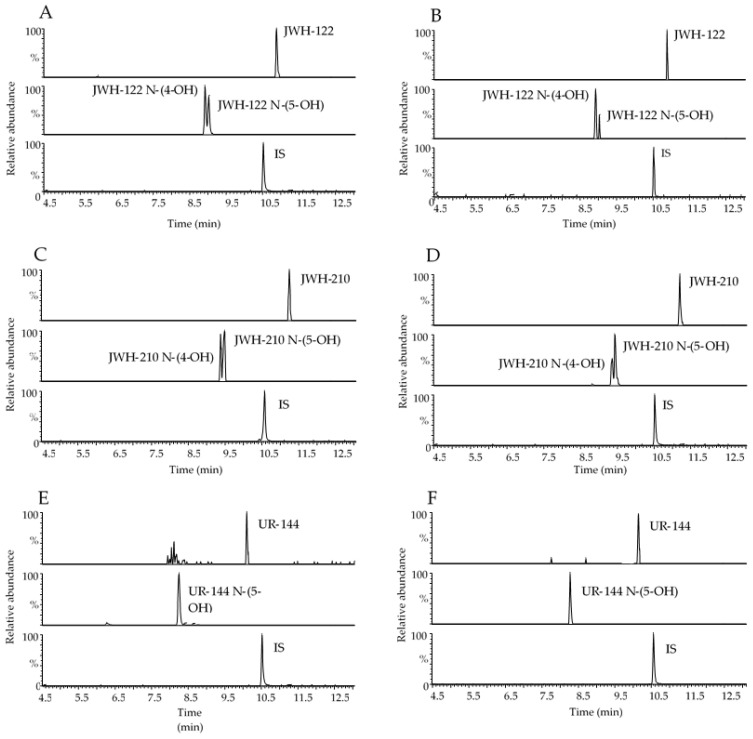
Representative range-mass UHPLC-HRMS chromatograms of 400 µL OF extract spiked with 0.5 ng/mL JWH-122, JWH-122 N-(4-OH) and JWH-122 N-(5-OH) (**A**), 0.5 ng/mL JWH-210, JWH-210 N-(4-OH) and JWH-210 N-(5-OH) (**C**), 0.5 ng/mL UR-144 and UR-144 N-(5-OH) (**E**); OF samples of consumers containing 3.14 ng/mL JWH-122, 0.29 ng/mL JWH-122 N-(4-OH) and JWH-122 N-(5-OH) in traces under the LOQ (**B**); 7.30 ng/mL JWH-210, 0.29 ng/mL JWH-210 N-(4-OH) and 0.66 ng/mL JWH-210 N-(5-OH) (**D**); 6.81 ng/mL UR-144 (**F**). IS, internal standard.

Representative chromatograms obtained following the extraction of 400 µL blank OF samples spiked with 1 and 0.5 ng/mL analytes under investigation for GC-MS and UHPLC-HRMS methods, respectively are reported in Figure 1 and Figure 2 together with chromatograms of real samples of consumers at 20 min after starting SCs smoking.

Linear calibration curves for SCs and their metabolites in OF samples showed determination coefficients (R^2^) equal or higher than 0.991 both for GC-MS and UHPLC-HRMS analysis. The methods were linear for all analytes from limit of quantification (analytes LOQ range: 0.5–2.3 ng/mL for GC-MS and 0.07–0.25 ng/mL for UHPLC-HRMS) to 50 ng/mL OF. Limits of detection (LODs) and of quantification (LOQs) fitted for the purposes of the study. Mean analytical recoveries obtained for the three different quality control (QC) samples were always above 80% except for UR-144 whose recovery was about 70%, applying both methodologies (Table 1).

The intra- and inter-assay precision (measured as coefficient of variation, %CV) and accuracy (measured as % Error) determined in the three QC samples (n = 15) showed values within ± 20% (Table 2).

No endogenous substances interfered with the detection of the analytes under investigation both in GC-MS and UHPLC-HRMS. No significant ion suppression/enhancement (less than 15%) occurred during GC-MS or UHPLC-HRMS runs. None of the analytes under investigation showed relevant degradation after three freeze/thaw cycles and concentrations were always within 10% initial one. No carry-over was observed in blank OF samples after injecting highest curve calibrators. To assess the concordance between GC-MS and UHPLC-HRMS methods, quantitative results for parent SCs in OF obtained with the two assays were compared. A good and statistically significant correlation was obtained when comparing the results from the two assays (*r*^2^ = 0.908 and Spearman’s rs 0.936, *p* < 0.0001). A non-significant bias (−0.013) was found using Bland-Altman plot.

### 2.2. Pharmacokinetics of JWH-122, JWH-210 and UR-144 and Their Metabolites

**Figure 3:**
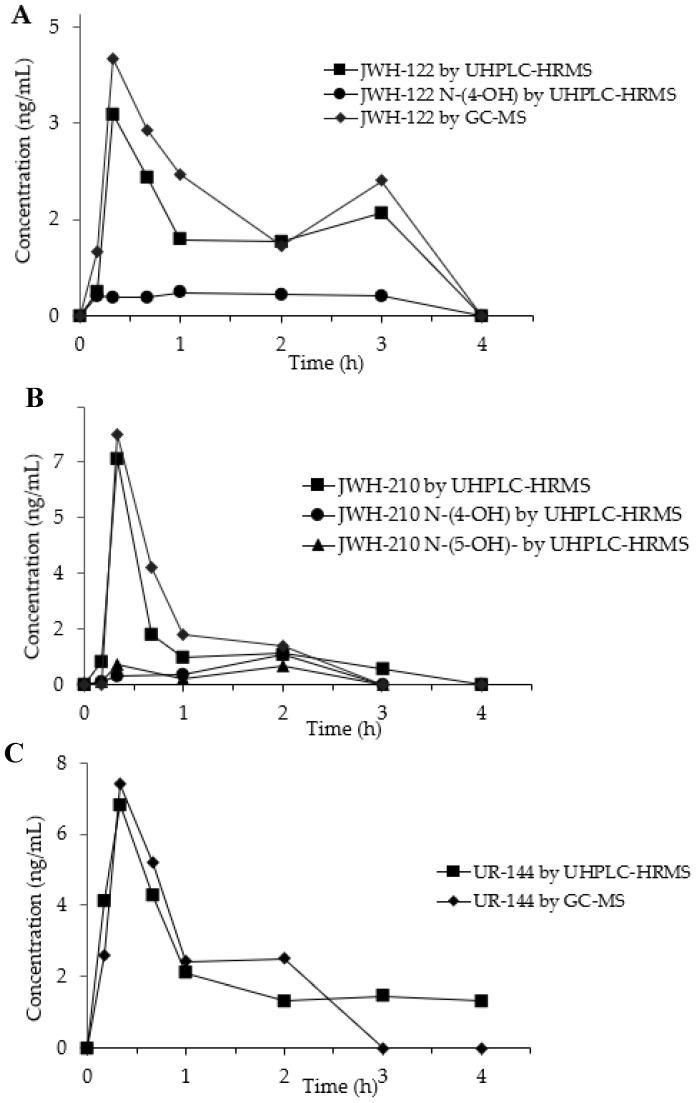
Concentration time-profiles of JWH-122 (**A**), JWH-210 (**B**), UR-144 (**C**) and their metabolites in OF of SCs consumers by GC-MS and UHPLC-HRMS.

SCs and their metabolites concentration–time curves in OF from the three consumers are depicted in Figure 3A–C.

In the OF sample from JWH-122 consumer, the parent drug presented a peak concentration (C_max_) 4.00 ng/mL (GC-MS) and of 3.14 ng/mL (UHPLC-HRMS) at 20 min (T_max_) after the start of SCs smoking. The concentration decreased at 3 h after SCs smoking and then a second peak was observed both in GC-MS (2.00 ng/mL) and UHPLC-HRMS (1.60 ng/mL) analyses. JWH-122 N-(4-OH) was quantified by UHPLC-HRMS showing a concentration range of 0.29–0.36 ng/mL between 10 min and 3 h after the smoking while JWH-122 N-(5-OH) was only detected in traces under the LOQ.

The concentration-time curves of the JWH-210 in consumer OF displayed after 20 min a peak concentration of 8.10 ng/mL 7.30 ng/mL by GC-MS and UHPLC-HRMS and, respectively. Then the SC decreased becoming undetectable after 3 h. JWH-210 N-(4-OH) and JWH-210 N-(5-OH) OF concentrations, measured by UHPLC-HRMS, peaked after 20 min (C_max_ 0.29 ng/mL and 0.66 ng/mL, respectively) and after 2 h (C_max_: 0.98 ng/mL and 0.61 ng/mL, respectively) following SC smoking.

The time peak of UR-144 occurred at 20 min from smoking start with C_max_ of 7.40 ng/mL by GC-MS and 6.81 ng/mL by UHPLC-HRMS and then it decreased to undetectable values within the next 4 h. UR-144 N-(5-OH) was only detected in traces during all the time-course and could never be quantified above the LOQ.

## 3. Discussion

SCs along with synthetic cathinones remain the most consumed new psychoactive substances (NPS) accounting for 28 and 36% seizures along Europe according to the European Monitoring Centre for Drugs and Drug Addiction [3]. Both classes of compounds present potent and long-lasting effects but also severe acute and chronic toxicity possibly leading to fatalities [4,10,30,31]. Hence, due to their potential health hazard, identification and quantification in conventional and non-conventional biological samples of consumers can be strictly important. Specifically, OF is a good alternative to blood for non-invasively SCs recent use monitoring.

We here present, for the first time, two complementary analytical methods for the determination of JWH-122, JWH-210 and UR-144 in OF of consumers. As reported above, we focused on these particular substances due to their recent spread on the Spanish illegal market [26].

The two methods based on different chromatographic characteristics resulted to be good complementary approaches for the analysis of SCs in OF. The last generation GC-MS system allowed the target determination of JWH-122, JWH-210 and UR-144, UHPLC-HRMS confirmed the presence of parent compounds determined by GC-MS and allowed the determination of hydrophilic metabolites, undetectable for their physico-chemical characteristics by GC-MS. It has to be added that since for NPS the lack of analytical standards is frequent, especially in case of metabolites, HRMS could have allowed metabolites identification even in absence of reference standards through the exact mass measurement and a comprehensive spectra library, such as the mzCloud mass spectral one. With UHPLC-HRMS, we could demonstrate that also metabolites of SCs under investigation are excreted in OF in low concentrations.

Analytically, a methodological limitation resided in the fact that, if used alone last generation GC-MS was not able to determine SCs hydrophilic metabolites in OF and due to matrix endogenous possible interferences GC-MS required a longer run-time. On the contrary, an analytical strength was due to the UHPLC-HRMS ability to detect and quantify SCs metabolites in OF even at very low concentration within few minutes of run time, thus with a shorter run time. The two methods thanks to the ease, cost-effectiveness, robustness and fast sample preparation could be used for a complete pharmacokinetic study and on a large sample size by the Spanish investigation group.

Even if few real samples were made available to prove analytical methods robustness Our results were consistent with previous pharmacokinetic studies investigating the presence of JWH-210 and JWH-018 in OF of consumers [24,25]. Furthermore, in the study on JWH-018 OF concentration after smoking two types of herbal products [25] SC peaked at 20 min and then decreased to baseline showing a similar OF pharmacokinetic profile to that of JWH-122, JWH-210 and UR-144, here presented.

Analytical data obtained from toxicological and forensic analyses are striking in the global challenge to health risks caused by new psychoactive substances [32].

## 4. Materials and Methods

### 4.1. Chemicals and Reagents

The SCs (4-methyl-1-naphthalenyl)(1-pentyl-1*H*-indol-3-yl)-methanone (JWH-122), (4-ethyl-1-naphthalenyl)(1-pentyl-1*H*-indol-3-yl)-methanone (JWH-210) and (1-pentyl-1*H*-indol-3-yl)(2,2,3,3-tetramethylcyclopropyl)-methanone (UR-144) and their respective metabolites (1-(4-hydroxypentyl)-1*H*-indol-3-yl)(4-methylnaphthalen-1-yl)methanone (JWH-122 N-(4-OH), (1-(5-hydroxypentyl)-1*H*-indol-3-yl)(4-methylnaphthalen-1-yl)-methanone (JWH-122 N-(5-OH), (4-ethylnaphthalen-1-yl)(1-(4-hydroxypentyl)-1*H*-indol-3-yl)methanone (JWH-210 N-(4-OH), (4-ethylnaphthalen-1-yl)(1-(5-hydroxypentyl)-1*H*-indol-3-yl)methanone (JWH-210 N-(5-OH) and [1-(5-hydroxypentyl)-1*H*-indol-3-yl](2,2,3,3-tetramethylcyclopropyl)-methanone (UR-144 N-(5-OH) were purchased from Cayman Chemical (Ann Arbor, MI, USA). Deuterated naphthalen-1-yl-(1-pentylindol-3-yl)- 1,1,2,2,3,3,4,4,5,5,5-D11-methanone (JWH-018-d_11_) used as internal standard was supplied by Lipomed (Milan, Italy). N,OBis(trimethylsilyl)trifluoroacetamide solution (BSTFA + 1%TMS) for derivatization was purchased from Restek Corporation (Bellefonte, PA, USA). Ultrapure water, methanol, acetonitrile (all UHPLC–MS/MS grade) and all other reagents (HPLC grade) were obtained from Carlo Erba (Milan, Italy).

### 4.2. Calibrators and Quality Control Solutions

Stock solutions of each analyte at 10 µg/mL, 1 µg/mL, 0.1 µg/mL and 0.01 µg/mL were prepared in methanol. Standard stock solutions containing JWH-018-d_11_ were prepared in methanol at 10 and 0.1 µg/mL for GC-MS and UHPLC-HRMS analyses, respectively. Stock solutions were stored in glass vials at −20 °C.

Drug-free OF samples were collected from laboratory staff, individually analysed during method validation to exclude any source of chromatographic interference and mixed to obtain a homogeneous pool of blank samples to be used for calibration standards and QC samples. GC-MS calibration points ranged from LOQ of each analyte, to 5 ng/mL, 10 ng/mL and 50 ng/mL while UHPLC-HRMS calibrators ranged from LOQ of each analyte, 0.5 ng/mL, 1 ng/mL, 5 ng/mL, 10 ng/mL and 50 ng/mL. Calibration curves were prepared daily for each analytical batch by adding the proper amounts of working solutions to 400 µL of pre-checked drug-free OF pooled sample. QC samples were prepared fortifying drug-free OF samples with suitable amounts of methanol SCs and metabolites working solutions. The same high and medium QC concentrations of 40 ng/mL and 20 ng/mL were used to establish intra-day and inter-day precision and accuracy for both GC-MS and UHPLC-HRMS assays while low QC were prepared at a concentration one and half times the calculated LOQ of each analyte for both analytical techniques.

### 4.3. Subjects, Study Design and Samples Collection

OF samples were donated by three synthetic cannabinoid consumers, who attended a private cannabis-smoking club in Spain, on February 2019, when SCs smoking in such clubs was allowed. They were two females (ages 33 and 25 years old, weights 54 and 67 kg, heights 1.50 and 1.71 m) and one male (39 years old, weight 60 kg and height 1.67 m). All three subjects reported a long cannabis consumption experience (from weekly to monthly use) and, at least, two previous uses of SCs, psychostimulants and hallucinogens. Each participant self-administered a cigarette containing SCs mixed with tobacco smoking in 5 min time. They selected the synthetic cannabinoid to be consumed in the session (the first female: JWH-122 1 mg, the second female: JWH-210 3 mg and the male: UR-144 1 mg), which was obtained from an unknown source, but analysed for content by a Drug Checking Service performed by a Spanish non-governmental organization (Energy Control, Barcelona, Spain). The doses were selected from previous experiences and in the range of those usually recommended in web fora and surveys. At the time of the study, these synthetic cannabinoids were not illegal in Spain and personal use was allowed in house and private clubs for cannabis smoking. Sample collection was authorized by the local Human Research Ethics Committee (CEI-HUGTiP ref. PI-18-267, Badalona, Spain). OF was obtained at baseline, 10, 20, 40 min and at 1, 2, 3 and 4 h after self-administration in Salivette tubes, that were then centrifuged, storing supernatants at −20 °C until analysis.

### 4.4. Synthetic Cannabinoids and Their Metabolites Extraction and Determination

An amount of 400 μL OF samples were extracted by adding 100 μL 0.1 M phosphate buffer pH 3.0 (acid pH was adjusted using drops of 1 N HCl), 20 μL of IS working solutions for UHPLC-HRMS analysis and 5 μL for GC-MS analysis. The samples were extracted twice with 3.0 mL of hexane:ethyl acetate mixture (9:1, *v*/*v*). The tubes were centrifuged at 4000 rpm for 4 min. The organic phase was transferred to clear tube and dried under nitrogen stream. In GC-MS analysis the dried samples were derivatized with 50 µL mixture of BSTFA containing 1% TMCS and acetonitrile (1:1, *v*/*v*) at 70 °C for 30 min. A volume of 1 μL was injected into the GC-MS system. The dried samples were resuspended with 100 μL mixture of mobile phase A (ammonium formate 2 mM in water, 0.1% formic acid) and B (ammonium formate 2 mM in methanol/acetonitrile 50/50, 0.1% formic acid) (50:50, *v*/*v*) and 10 μL were injected into the UHPLC-HRMS instrument.

### 4.5. GC-MS Instrumentation

Analyses were performed with an Intuvo 9000 GC System (Agilent Technologies, Palo Alto, CA, USA) coupled to a 5977 B MSD (Agilent Technologies, Palo Alto, CA, USA). Extracted samples were injected in splitless mode at 260 °C into the Ultra-Inert Intuvo GC column (HP-5MS UI, 30 m × 250 µm i.d, film thickness 0.25 µm; Agilent Technologies). Helium was used as carrier gas working at a flow rate of 1.2 mL/min. The oven ramp temperature was programmed as follows: initial temperature of 70 °C held for 2 min and increased at 30 °C/min to 190 °C, then increased to 290 °C at 5 °C/min for 10 min. Subsequently, the temperature was increased at 40 °C/min to 340 °C to eliminate impurities from the column. The transfer line temperature was set at 320 °C. Mass spectrometer with positive ionization operated in selected ion monitoring mode (SIM).

The characteristic ions for the SIM mode were chosen using international libraries such as NIST Research Library (National Institute of Standards and Technology) and SWGDRUG Library version 3.6. GC-MS analytes retention time, quantitative and qualitative ions are reported in Table 3.

### 4.6. UHPLC-HRMS Instrumentation

The UHPLC/ESI Q-Orbitrap system was equipped with an Ultimate 3000 LC pump and an Ultimate 3000 autosampler, coupled to a Q Exactive Focus mass spectrometer (ThermoFisher Scientific Bremen, Germany). The Q Exactive with a heated electrospray ionization (HESI) probe operated in positive ionization mode and was controlled by by Xcalibur 2.2 software (ThermoFisher Scientific, Bremen, Germany). A Kinetex Biphenyl 100A (100 × 2.1 mm, 2.6 µm) (Phenomenex, Milan, Italy) was used for the chromatographic separation. The run time lasted 18 min with a mobile phase flow rate at 0.6 mL/min. Mobile phases were ammonium formate 2 mM in water with 0.1% formic acid (mobile phase A) and ammonium formate 2 mM in methanol/acetonitrile 50:50 (*v*/*v*) with 0.1% formic acid (mobile phase B). The UHPLC gradient profile started with an initial condition of 20% B, held for 2 min, increased to 81.4% B within 9 min, increased to 100% B within 0.2 min, held for 4.3 min, then returned to initial condition within 0.1min, and finally held for 2.4 min. LC flow was directed to waste for the first 4.5 min and after 13.5 min. Autosampler and column oven temperatures were kept at 4 °C and 40 °C, respectively. MS parameters have been set as follows: ionization voltage 3.0 kV; sheath gas and auxiliary gas 35 and 15 arbitrary units, respectively; S-lens radio frequency RF level was 60; vaporizer temperature and capillary temperature were 320 °C. Nitrogen was used for spray stabilization, for collision induced dissociation experiments in the higher-energy collisional dissociation (HCD) cell and as damping gas in the C-trap. Every week the instrument was tuned in positive and negative modes. Data were acquired in full-scan data-dependent MS^2^ (dd-MS^2^) mode with an inclusion list containing the exact masses of over 1400 compounds including parent compounds and their metabolites. The full MS scan range was 50–650 *m*/*z*, data were acquired with a mass resolution of 70,000. The precursor ions were fragmented with stepped normalized collision energy of 30, 35.0, 52.5 V and Orbitrap analyzer had a resolution of 17,500 and an isolation window of 2.0 *m*/*z*. Full scan data files were processed by Thermo Scientific XCalibur software 3.2 and mzCloud Mass Spectral Library was used as mass spectra international library for peak identification (Advanced Mass Spectral Database; www.mzcloud.org). This specific software uses a built-in database and mass spectral library with more than 1400 substances to identify drugs and metabolites by comparing retention times, isotope pattern matching and elemental composition determinations of compounds. UHPLC-HRMS analytes retention time, quantitative and qualitative ions are reported in Table 3.

### 4.7. Method Validation

The analytical methods, prior to real samples analysis, were completely validated applying the internal protocol and in accordance to the internationally established criteria [33,34] for the assessment of linearity, LOD, LOQ, precision, accuracy, carry-over, matrix effect, ion suppression, recovery and stability. LOD was defined as the lowest analyte concentration that can be detected and identified. A signal-to-noise ratio higher than 3 was used for LOD measurement analysing different blank OF samples with decreasing concentrations of the spiked analyte. LOQ was the lowest concentration with a signal-to-noise ratio higher than 10. Validation parameters were calculated using five different daily replicates of three QC samples (low, medium, and high) along five subsequent working days. The effect of three freeze-thaw cycles (storage at −20 °C) on the compounds stability in OF was evaluated by repeated analysis of five replicates of the three QC samples. Matrix effect and recovery were determined using the experimental plan proposed by Matuszewski et al. [35]. Set 1 was five replicates of pure analytical standards QC solutions. Sets 2 and 3 were five different replicates of blank samples fortified with QC solutions after and before extraction, respectively. Matrix effect was determined by dividing mean peak areas of set 2 by set 1 multiplied by 100, while recovery was determined by comparing the mean peak areas of compounds under investigation obtained in set 3 to those in set 2 multiplied by 100. Evaluation of carry-over was performed by injecting high calibrator followed by analysing blank OF samples.

## Figures and Tables

**Table 1:** Calibration curve parameters, LODs, LOQs and recovery of SCs and their metabolites in OF by GC-MS and UHPLC-HRMS.

**GC-MS**						
**Analytes**	**Correlation Coefficient (R^2^) ^a^**	**LOD** **(ng/mL)**	**LOQ** **(ng/mL)**	**Mean Recovery (%) ^b^**		
				**QC Samples**		
				Low	Medium	High
JWH-122	0.997 ± 0.003	0.30	0.50	80.3	81.8	81.5
JWH-210	0.996 ± 0.001	0.70	1.00	83.7	83.9	84.4
UR-144	0.997 ± 0.003	0.70	2.30	71.5	78.0	71.1
**UHPLC-HRMS**						
**Analytes**	**Determination Coefficient (R^2^) ^a^**	**LOD** **(ng/mL)**	**LOQ** **(ng/mL)**	**Mean Recovery (%) ^b^**		
				**QC Samples**		
				**Low**	**Medium**	**High**
JWH-122	0.997 ± 0.003	0.07	0.25	84.4	93.5	98.6
JWH-122 N-(4-OH)	0.996 ± 0.001	0.03	0.10	97.4	84.5	97.8
JWH-122 N-(5-OH)	0.997 ± 0.003	0.03	0.10	85.3	87.6	88.8
JWH-210	0.997 ± 0.004	0.06	0.20	92.8	85.7	97.4
JWH-210 N-(4-OH)	0.996 ± 0.001	0.02	0.07	90.6	87.0	101.5
JWH-210 N-(5-OH)	0.996 ± 0.004	0.03	0.10	89.9	86.8	99.8
UR-144	0.991 ± 0.009	0.05	0.15	71.8	75.3	70.1
UR-144 N-(5-OH)	0.996 ± 0.003	0.03	0.10	97.9	102.1	101.2

^a^ Mean of three replicates of calibration curves; ^b^ Mean of five replicates; LOD, limits of detection; LOQ, limits of quantification.

**Table 2:** Intra- and inter-day precision and accuracy values for SCs and their metabolites in OF by GC-MS and UHPLC-HRMS.

**GC-MS**									
**Analytes**	**Intra-Day Precision** **(CV%)**			**Inter-Day Precision** **(CV%)**			**Accuracy** **(% Error)**		
**QC Samples ^a^**									
	**Low**	**Medium**	**High**	**Low**	**Medium**	**High**	**Low**	**Medium**	**High**
JWH-122	4.8	7.3	9.4	9.7	8.7	6.9	8.7	8.7	7.3
JWH-210	3.2	9.1	5.3	9.9	5.3	7.2	9.9	10.2	10.1
UR-144	5.7	9.2	8.3	10.1	12.3	11.2	9.1	8.5	9.9
**UHPLC-HRMS**									
**Analytes**	**Intra-Day Precision** **(CV%)**			**Inter-Day Precision** **(CV%)**			**Accuracy** **(% Error)**		
**QC Samples ^a^**									
	**Low**	**Medium**	**High**	**Low**	**Medium**	**High**	**Low**	**Medium**	**High**
JWH-122	2.2	4.2	8.6	11.4	5.7	5.9	13.7	9.9	7.7
JWH-122 N-(4-OH)	4.1	6.9	3.1	10.1	8.3	7.4	10.6	9.6	3.7
JWH-122 N-(5-OH)	7.6	15.4	12.8	12.0	15.4	11.9	8.7	7.5	13.5
JWH-210	6.2	5.2	8.4	15.6	5.4	6.9	8.5	9.8	4.2
JWH-210 N-(4-OH)	4.8	7.1	6.2	12.6	7.1	5.2	11.4	8.7	10.3
JWH-210 N-(5-OH)	15.1	7.7	8.5	17.4	11.2	8.4	8.0	10.3	10.4
UR-144	7.6	15.4	12.8	12.0	15.4	11.9	8.7	7.5	10.8
UR-144 N-(5-OH)	4.1	6.9	3.8	10.1	8.0	7.0	10.6	9.6	3.7

^a^ Mean of five replicates (*n* = 15) along five subsequent working days.

**Table 3:** UHPLC-HRMS and GC-MS MS parameters and relative retention time (RRt) in targeted MS/MS and SIM mode, respectively.

UHPLC-HRMS					GC-MS		
Analytes	Chemical Formula	RRt (min)	Quantifier (*m*/*z*) [M + H]^+^	Qualifiers (*m*/*z*)	RT (min)	Quantifier (*m*/*z*)	Qualifiers (*m*/*z*)
JWH-122	C_25_H_25_NO	1.03	356.2008	169.0646 214.1223	13.9	298	214 284
JWH-122 N-(4-OH)	C_25_H_25_NO_2_	0.84	372.1949	141.0698 169.0649	-	-	-
JWH-122 N-(5-OH)	C_25_H_25_NO_2_	0.85	372.1951	141.0698 169.0647	-	-	-
JWH-210	C_26_H_27_NO	1.06	370.2157	183.0803 214.1222	14.8	214	144 183
JWH-210 N-(4-OH)	C_26_H_27_NO_2_	0.88	386.2105	144.0443 183.0806	-	-	-
JWH-210 N-(5-OH)	C_26_H_27_NO_2_	0.89	386.2106	183.0803 230.1172	-	-	-
UR-144	C_21_H_29_NO	0.96	312.2325	125.0961 244.0963	7.1	214	144 296
UR-144 N-(5-OH)	C_21_H_29_NO_2_	0.78	328.2271	125.0961 230.1172	-	-	-
JWH-018-d_11_	C_24_H_12_D_11_NO	1.00	353.2537	-	11.99	352	-

## Abbreviations

SCs	Synthetic Cannabinoids
NPS	New psychoactive Substances
GC-MS	Gas chromatography-mass spectrometry
UHPLC-HRMS	Ultra-high performance liquid chromatography-high-resolution mass spectrometry
OF	Oral Fluid
JWH-122	(4-methyl-1-naphthalenyl)(1-pentyl-1H-indol-3-yl)-methanone
JWH-122 N-(-4-OH)	(1-(4-hydroxypentyl)-1H-indol-3-yl)(4-methylnaphthalen-1-yl)-methanone
JWH-122 N-(-5-OH)	(1-(5-hydroxypentyl)-1H-indol-3-yl)(4-methylnaphthalen-1-yl)-methanone
JWH-210	(4-ethyl-1-naphthalenyl)(1-pentyl-1H-indol-3-yl)-methanone
JWH-210 N-(4-OH)	(4-ethylnaphthalen-1-yl)(1-(4-hydroxypentyl)-1H-indol-3-yl)-methanone
JWH-210 N-(5-OH)	(4-ethylnaphthalen-1-yl)(1-(5-hydroxypentyl)-1H-indol-3-yl)-methanone
UR-144	(1-pentyl-1H-indol-3-yl)(2,2,3,3-tetramethylcyclopropyl)-methanone
UR-144 N-(5-OH)	1-(5-hydroxypentyl)-1H-indol-3-yl](2,2,3,3-tetramethylcyclopropyl)-methanone
R^2^	Determination coefficient
LOD	Limit of detection
LOQ	Limit of quantification
QC	Quality control
CV	Coefficient of variation
C_max_	Maximum concentration
T_max_	Time to reach the maximum concentratio
IS	Internal standard
